# Assessing and decomposing inequality of opportunity in access to child health and nutrition in sub-Saharan Africa: evidence from three countries with low human development index

**DOI:** 10.1186/s12939-020-01258-5

**Published:** 2020-08-25

**Authors:** Yacobou Sanoussi, Bright Opoku Ahinkorah, Aduragbemi Banke-Thomas, Sanni Yaya

**Affiliations:** 1 University of Kara, Faculty of Economics and Management (FaSEG), Kara, Togo; 2 The Australian Centre for Public and Population Health Research, Faculty of Health, University of Technology Sydney, Sydney, NSW Australia; 3 LSE Health, London School of Economics and Political Science, London, UK; 4 School of International Development and Global Studies, Faculty of Social Sciences, University of Ottawa, 120 University Private, Ottawa, ON K1N 6N5 Canada; 5 The George Institute for Global Health, The University of Oxford, Oxford, UK

**Keywords:** Inequality of health opportunities, Human opportunity index, Dissimilarity index, Maternal and child health, Congo, Guinea Bissau, Mali

## Abstract

**Background:**

Inequality of opportunity in health and nutrition is a major public health issue in the developing regions. This study analyzed the patterns and extent of inequality of opportunity in health and nutrition among children under-five across three countries sub-Saharan Africa with low Human development index (HDI).

**Methods:**

We used data from the Multiple Indicator Cluster Survey of the Democratic Republic of Congo (20,792 households, 21,756 women aged 15 to 49 and 21,456 children under five), Guinea Bissau (6601 households, 10,234 women aged 15–49 and 7573 children under five) and Mali (11,830 households, 18,409 women in 15–49 years and 16,468 children under five) to compute the human opportunity index (HOI) and the dissimilarity index (D-index). Secondly, the Shapley decomposition method was used to estimate the relative contribution of circumstances that are beyond the control of children under-five and affecting their development outcomes in later life stages.

**Results:**

The study revealed that children belonging to the most favorable group had higher access rates for immunization (93.64%) and water and sanitation facilities (73.59%) in Guinea Bissau. In Congo DR, the access rate was high for immunization (93.9%) for children in the most favorable group. In Mali, access rates stood at 6.56% for children in the most favorable group. In Guinea Bissau, the inequality of opportunity was important in access to health services before and after delivery (43.85%). In Congo DR, the inequality of opportunity was only high for the immunization composite indicator (83.79%) while in Mali, inequality of opportunity was higher for access to health services before and after delivery (41.67%).

**Conclusion:**

The results show that there are efforts in some places to promote access to health and nutrition services in order to make access equal without distinction linked to the socio-economic and demographic characteristics in which the children live. However, the inequalities of opportunity observed between the children of the most favorable group and those of the least favorable group, remain in general at significant levels and call on government of these countries to implement policies taking them into account.

## Background

Child health and nutrition are crucial factors in child development, both of which have capacity to predict the trajectory towards health outcomes in adulthood. Specifically, malnutrition, in the forms of stunting and wasting, has been shown to delay cognitive, motor, and social development, which can predicate a multitude of adverse effects in adulthood [1–3]. Globally, about 7.7% of children were wasted, 24.5% were stunted and 15% were underweight in 2015. Africa and South-East Asia have reported the highest prevalence of undernutrition, with the African region accounting for about 39.4% of stunting, 24.9% of the underweight and 10.3% of wasting among children under-five [4, 5]. In Africa, poverty has been identified as one of the major causes of malnutrition [6–8]. A recent joint report by UNICEF and the World Bank says that about 50% of children in sub-Saharan Africa are living in extreme poverty and they contribute to over 51% of the world’s extremely poor children [9]. Hence, malnutrition is more likely to occur in countries like the Democratic Republic of Congo, Guinea Bissau and Mali, who have been considered as the top three countries in sub-Saharan Africa with the lowest Human development index (HDI).

Inequalities in the distribution of external inputs and circumstances that are out of children’s control, including household wealth and residence, as well as the availability and quality of child health services, food, clean water, and sanitation, result in inequality of opportunity (IoP) for disadvantaged children [10, 11]. IoP in health and nutrition is a significant problem in the developing regions such as sub-Saharan Africa [12, 13], where unfavorable deficits and inequalities in early life contribute to inequality in child health and nutrition outcomes [14, 15].

Numerous studies have examined the relationship between unequal circumstances and disparities in child health and nutrition outcomes in various developing countries with studies across Asia and Africa identifying differences in parental wealth as a circumstance that causes considerable IoP in nutrition and child health outcomes [16–23]. Lack of clean water and poor sanitation have also been identified as contributors to inequalities in child health [17, 24]. Differences in mother’s education and residence are other principal determinants that impact IoP in child health and nutrition [17, 19, 23]. Other factors that have been identified include religion and mother’s race [17, 25, 26].

All the above-mentioned circumstances which are beyond the control of children under-five strongly influence their ability to develop and lead productive lives. Given the direct impact of differences in circumstances on IoP and the significance of equality of opportunity on children’s health and nutrition, there is a need to identify the patterns and extent of IoP in health and nutrition outcomes of children. By extension, decomposing IoP helps to identify and quantify the impact of each examined circumstance in explaining inequalities. The analysis reflects how far the sub-Saharan African countries are, from providing fair and equal access to a set of critical child health and nutrition development outcomes, irrespective of encountered circumstances. Accordingly, this study has two objectives: first, to analyze the patterns and extent of IoP in health and nutrition among children under-five in the Democratic Republic of Congo, Guinea Bissau and Mali. Secondly, to use the Shapley decomposition method to estimate the relative contribution of circumstances that are beyond the control of children under-five and affecting their development outcomes in later life stages.

## Materials and methods

### Data sources

We used data from the most recent Multiple Indicator Cluster Survey (MICS) of three countries (Democratic Republic of Congo, Guinea Bissau and Mali) in sub-Saharan Africa. These countries were selected based on the fact that they are at the top in sub-Saharan Africa with the lowest HDI and for which data are available for the period 2014–2019.

As an international household survey program, the MICS program was developed by UNICEF from 1990 and aims to support countries in the collection of internationally comparable data on a wide range of indicators. Among other things, the MICS provides: (i) detailed information for the assessment of the situation of children and women (children's nutritional status, women's fertility history, water and sanitation, characteristics of household; (ii) basic data to assess the Millennium Development Goals (MDGs) and monitor the Sustainable Development Goals (SDGs) [27].

For the Democratic Republic of Congo, the sixth round of MICS conducted in 2017–2018 concerned a sample size of 20,792 households, representative at the national level, for both urban and rural areas and at the level of twenty-six provinces of the country. Information is also collected from 21,756 women and 6113 men aged 15 to 49 and 21,456 children under five. In Mali, data from the fifth round of MICS conducted in 2015 were collected from 11,830 households with a coverage rate of 99.8%. It is a representative survey of the population which provides detailed information on 18,409 women and 7430 men in 15–49 years and 16,468 children under five. The Guinea-Bissau MICS-5 conducted in 2014 from 6601 households and provides information on 10,234 women and 4232 men aged 15–49 and 7573 children under five.

### Variables and measurements

#### Access to healthcare variables

Based on insight from Ersado and Aran [28] study, we selected seven variables for the analysis of inequalities in healthcare outcomes. These are: *i. Antenatal care or prenatal care (women’s routine health control during pregnancy); ii. Birth’s place (birth in health facilities or elsewhere); iii. Birth attended (assisted birth skilled health personnel or others); iv. Child’s postnatal check-up (children medical checkup after delivery); v. Regular immunizations within 1 year after birth (vaccines to protect children from diseases); vi. Access to safe water (water from official sources of drinking water); vii. Access to toilet (availability of toilet in the house)*. These variables were used to construct three composite healthcare outcome indicators. The first indicator (HA1) relates to access to healthcare services before and after birth. This indicator was constructed by considering that children have access to healthcare services before and after birth if, at the same time, the mother received medical check-ups during pregnancy, gave birth in a health center assisted by qualified medical personnel and the child received postnatal check-up. The second (HA2) concerns access to immunizations. This indicator was built using the same information as that of the variable *immunizations*. The third indicator (HA3) considers access to housing services (water and sanitation facilities). This indicator was obtained by considering that an individual has access to housing services if, at the same time, he uses water from official sources of drinking water and has *toilet in the house in which he/she lives.*

#### Nutrition outcome variables

In order to explore the levels and trends in malnutrition and micronutrient intake, we selected nutrition outcome variables such as: blood tests (*blood samples taken for the purpose of the assessment of nutritional status)*, stunting, wasting and underweight. The last three are related to common anthropometric measures [22, 29] and were used to construct a composite indicator noted NUT1. This indicator (NUT1) is related to the growth status of children. It is constructed by considering that children have good growth status if, at the same time, all the anthropometric indicators are normal. The second one, is noted NUT2 and is related to the first nutrition outcome variable (*blood tests). This indicator (NUT2) was obtained by using information related to blood tests.*

*We consider that children have access to nutrition outcome or opportunity if they have good growth status or a blood sample has been taken from them for the purpose of the assessment of nutritional status.*

*In this research work, the opportunities are related to the two groups of variables above (healthcare and nutrition). The idea is to consider that the available services relating to health and nutrition are opportunities for children’s health. But access to these opportunities is influenced by certain characteristics that are beyond the control of these children.*

#### Circumstances variables

For each country, we retained the same variables of circumstances which are determinants of the health and nutritional status of children. A total of seven circumstance variables were considered: (*i) residence area; (ii) gender of child; (iii) number of children under 5; (iv) age of household head; (v) father’s education; (vi) mother’s education; (vii) economic wellbeing.* These circumstance variables were then used to subdivide the sample of children into several sub-groups ranging from the most favorable to the least favorable group. The objective here was to bring together children with identical life circumstances in order to capture the influence of the gap between the different groups on access to healthcare and nutritional outcome. These groups are built from a set of circumstantial variables having the same effects on access to healthcare and nutritional outcome. The idea of building groups is to take into account the interactive effects of the different circumstantial variables and not their individual effects given, for example, that a child in a poor family in a rural area will not necessarily have the same difficulties than one who is in a poor family but in urban area. The rest of this article will focus much more on the two extreme sub-groups (the most and the least favorable group). Thus, the least favorable group is built with characteristics recognized in the economic literature as having negative effects on access to healthcare and nutritional outcome such as: rural areas, poor households with more than two children for which the head has a low level of education. The most favorable group is characterized by circumstances recognized as having positive effects on access to healthcare and nutritional outcome such as urban areas, rich households with at most two children for which the head has a high level of education.

### Analytical steps

To analyze the patterns and extent of inequality of opportunity in health and nutrition among children under-five, we used the methodological framework of some previous studies [30, 31] to compute the human opportunity index (HOI) and the dissimilarity index (D-index). The interest given to IoP in children lies in the fact that they do not constitute a decision center and cannot choose between having access or not to the variables of health and nutrition results. Also, policies to combat inequalities in childhood are more effective than those implemented later.

In the first step of this analysis, we define a binary variable as follows [30, 31]:

1 $$ {z}_i=\left\{\begin{array}{c}1\kern1.5em if\ the\ {i}^{th} child\  has\  access\ to\ health\ or\ nutrition\ opportunity\\ {}0\kern0.5em if\ not\end{array}\right. $$

It follows from the preceding expression that the probability that the *i*^*th*^ child i has access to the opportunities retained is given by:

2 $$ {p}_i=E\left({z}_i\right) $$

By considering the fact that this probability is influenced by the life circumstance variables which are out of the children’s control, Eq. 2 can be redefined by means of a simple logit model as follows:

3 $$ {p}_i=\frac{e^{\left({\beta}_0+{\sum}_{j=1}^k{\beta}_j{x}_{ij}\right)}}{1+{e}^{\left({\beta}_0+{\sum}_{j=1}^k{\beta}_j{x}_{ij}\right)}} $$

*K* is a set of circumstance variables: *x*_*ij*_, *x*_*i*1_, *x*_*i*2_, …, *x*_*ik*._

We used the maximum likelihood method to estimate the vector of parameters ***β***_***j***_ of the logit model and obtain the maximum likelihood estimate ${\hat{\boldsymbol{p}}}_{\boldsymbol{i}}$. The latter is an estimate of the probability of access depending on the selected variables of circumstances. From this probability, we can now determine the dissimilarity index which represents the inequality of opportunity. This index gives information on the dissimilarity of access rates to a given service or available opportunities. It is estimated as follows:

4 $$ \hat{D}=\frac{1}{2\overline{p}}{\sum}_{i=1}^n{w}_i\left|{\hat{p}}_i-\overline{p}\right| $$

$$ Where=\left\{\begin{array}{c}\hat{\boldsymbol{D}\ } is\ the\ estimated\ relative\ mean\ deviation\ \\ {}{\boldsymbol{w}}_{\boldsymbol{i}} is\ the\ population\ weight\ associated\ to\ the\ specific\ opportunity\\ {}\overline{\boldsymbol{p}\ } is\ \mathrm{is}\ \mathrm{the}\ \mathrm{average}\ \mathrm{prevalence}\ \mathrm{of}\ \mathrm{access}\ \mathrm{to}\ \mathrm{selected}\ \mathrm{services}\end{array}\right. $$

The average prevalence of access to services or opportunity selected, called level of coverage, is obtained as follows:

5 $$ \overline{p}={\sum}_{i=1}^n{w}_i{\hat{p}}_i $$

The dissimilarity index (D-index) which measures the level of inequality of opportunity depending on different circumstances ranges from 0 to 1 (0 to 100 in percentage terms), and takes the value zero in a situation when opportunities are equal in terms of benefits for each child. To measure equity of opportunity, we use the difference between the unit and the D-index. This difference is noted **E** and is given by the expression:

6 $$ E=\left(1-D\right) $$

#### Human opportunity index

The HOI is obtained by the following expression:

7 $$ HOI=\overline{p}\left(1-D\right) $$

This equation shows an inverse relationship between the HOI and the D-index. The latter takes values between 0 and 1. When it increases and becomes close to 1, the HOI decreases. Thus, an increase in the value of the human opportunity index (HOI) can be done by increasing both coverage ($\overline{p}\Big)$ and equity (E) or increasing only the coverage and decreasing the dissimilarity index.

*All these indices were calculated on the basis of the two extreme groups constructed as follows:*

$$ Groups=\left\{\begin{array}{c}1\ \left(\boldsymbol{Most}\ \boldsymbol{advantage}\ \boldsymbol{group}\right)\  if\ children\ live\ in\ \mathrm{urban}\ \mathrm{areas},\mathrm{rich}\ \mathrm{households}\ \mathrm{with}\ \mathrm{a}\mathrm{t}\ \mathrm{most}\ \mathrm{t}\mathrm{wo}\ \mathrm{children}\\ {}\mathrm{for}\ \mathrm{which}\ \mathrm{the}\ \mathrm{head}\ \mathrm{has}\ \mathrm{a}\ \mathrm{high}\ \mathrm{level}\ \mathrm{of}\ \mathrm{education}\\ {}2\ \left( Least\ \boldsymbol{advantage}\ \boldsymbol{group}\right)\  if\ children\ live\ in\ \mathrm{rural}\ \mathrm{areas},\mathrm{poor}\ \mathrm{households}\ \mathrm{with}\ \mathrm{more}\ \mathrm{than}\ \mathrm{t}\mathrm{wo}\ \mathrm{children}\\ {}\mathrm{for}\ \mathrm{which}\ \mathrm{the}\ \mathrm{head}\ \mathrm{has}\ \mathrm{a}\ \mathrm{low}\ \mathrm{level}\ \mathrm{of}\ \mathrm{education}\end{array}\right. $$

#### Decomposition of the inequality by the Shapley value

Following Shorrocks [32], we used Shapley decomposition method to estimate the contribution of each circumstance defined above to inequality in access to health and nutrition outcome variables selected. If we assume that the D-index and the HOI are influenced by the set of circumstance variables defined above, it is also important to emphasize that the increase in the circumstance variables can increase the dissimilarity index. This effect can be measured by the expression below:

8 $$ {D}_A={\sum}_{s\subseteq N\backslash \left\{A\right\}}\frac{\left|s\right|!\left(n-\left|s\right|-1\right)!}{n!}\left[D\left(S\cup \left\{A\right\}\right)-D(S)\right] $$

In this equation: **A** is the additional circumstance variable, *D*_*A*_ the impact of adding a circumstance A, **N** represents the set of the n circumstances, **S** is the subset of N circumstances obtained without the circumstance **A,** ***D***(***S***) is the dissimilarity index obtained with the set of circumstances *S* without the circumstance **A,** *D*(*S* ∪ {*A*}) is the dissimilarity index considering with the set of circumstances S and circumstance **A**.

The application of the shapely decomposition method allowed us to capture the contribution of each circumstance variable omitted to the dissimilarity index as follows:

9 $$ {\theta}_A=\frac{D_A}{D(N)} $$

In the previous expression, ${\sum}_{i\in N}{\theta}_A=1$ meaning that the contribution of all the variables of circumstance must amount to 1 (100%). All the steps of this methodological approach are used to achieve the objectives of this article.

## Results

Table 1 presents the descriptive statistics of the healthcare outcome variables for the three countries. It is clear from the table that access to these healthcare outcome variables exceeded 50% only for immunization and access to health for all three countries. For the rest, access is less than 50%, except the case of birth’s place (Congo DR and Mali) and antenatal care (Congo DR and Guinea Bissau). There was a significant disparity between countries according to the levels of access to healthcare variables with the exception of access to sanitation and antenatal care, where there was approximately 11% points and 9% points between the country with the highest level and the one with the lowest level.

**Table 1:** Descriptive Statistics of Healthcare Outcome Variables by countries

Health Outcome variables	Antenatal care (%)	Birth’s place (%)	Birth attended by Professionals (%)	Postnatal check (%)	Immunization (%)	Access to water (%)	Access to sanitation (%)
Countries							
Congo DR	53.22	73	49.90	33.87	86.79	26.64	83.76
Guinea Bissau	55.81	23.64	27.11	25.82	61.25	41.61	72.45
Mali	44.25	56.51	35.17	13.57	56.39	46.20	84.15

Source: Authors, based on MICS data

Table 2 shows that stunting in Congo DR, Guinea Bisssau and Mali were approximately 19%, 18% and 15% respectively. Similarly, underweight was approximately 17% in Mali, 16% in Congo DR and 11% in Guinea Bissau. With wasting, there was approximately 6% points between the country with the highest level (11.56%) and the one with the lowest level (6%).

**Table 2:** Descriptive Statistics of Nutrition Outcome Variables by countries

Nutrition Outcome variables	Blood tests (%)	Stunting (%)	Wasting (%)	Underweight (%)
Countries				
Congo Democratic Republic	68.58	19.24	5.6	15.72
Guinea Bissau	51.85	18.34	5.93	10.7
Mali	69.17	15.09	11.56	16.99

Source: Authors, based on MICS data

Note: the values in the column “Blood tests” are relative to the percentage of children whose mothers have had a blood sample

Table 3 reveals that with an overall circumstance score of 86.8%, Congo DR was the most advantaged country while Mali was the least advantaged group (5.98%).

**Table 3:** Descriptive Statistics of Circumstance Variables by countries: mean, standard deviation (in parenthesis), range (in brackets) for quantitative variables and percentage of the reference category for the dummy variables

Circumstance variables	Residence area	Gender of Child	Number of children 0–5	Age of household head	Father’s education	Mother’s education	Economic Wellbing	Most advantage Group
Countries								
Congo Democratic Republic	59.99	49.69	1.75 (0.97) [0–9]	43.37 (12.43) [17–94]	30.97	56.52	56.89	86.80
Guinea Bissau	79.51	47.47	2.73 (1.53) [1–10]	51.1 (14.31) [19–95]	81.28	86.04	81.65	13.58
Mali	83.83	49.72	3.24 (2.09) [1–14]	54.26 (15.02) [15–95]	83.25	82.67	64.67	5.98

Source: Authors, based on MICS data

Table 4 shows results on HOI on selected healthcare indicators by countries. HOI for the selected healthcare indicators in Congo DR, Guinea Bissau and Mali were 10.91%, 1.89% and 1.16%, respectively.

**Table 4:** Human Opportunity Index on Selected Healthcare Indicators by countries

		IHA1 (%)	Antenatal care (%)	Birth place (%)	Birth attended by professionals (%)	Postnatal check(%)	Immunizations (%)	IHA3 (%)	Access to water (%)	Access facilities (%)
**Coverage (C)**	**Congo DR**	13.34	46.06	72.80	43.13	33.71	86.78	26.41	27.89	85.16
	**Guinea Bissau**	3.36	43.03	21.32	20.16	19.83	62.47	40.42	44.58	75.89
	**MALI**	1.99	36.30	56.20	28.91	11.10	57.65	43.49	47.30	85.08
**Penalty (P)**	**Congo DR**	2.43	6.45	7.05	6.30	2.44	2.98	12.98	13.46	4.61
	**Guinea Bissau**	1.47	5.85	6.35	3.18	3.48	5.53	10.19	9.16	11.23
	**MALI**	0.83	3.34	10.34	3.67	1.21	3.92	9.55	8.18	7.61
**Dissimilarity (D)**	**Congo DR**	18.19	13.99	9.69	14.60	7.23	3.44	49.16	48.26	5.41
	**Guinea Bissau**	43.85	13.60	29.78	15.78	17.54	8.86	25.21	20.54	14.80
	**MALI**	41.67	9.20	18.40	12.71	10.88	6.81	21.96	17.30	8.94
**Equality (E)**	**Congo DR**	81.81	86.01	90.31	85.4	92.77	96.56	50.84	51.74	94.6
	**Guinea Bissau**	56.15	86.4	70.22	84.22	82.46	91.14	74.79	79.46	85.2
	**MALI**	58.33	90.8	81.6	87.29	89.12	93.19	78.04	82.7	91.06
**HOI**	**Congo DR**	10.91	39.62	65.75	36.83	31.27	83.79	13.43	14.43	80.56
	**Guinea Bissau**	1.89	37.18	14.97	16.97	16.36	56.92	30.23	35.43	64.66
	**MALI**	1.16	32.96	45.86	25.23	9.89	53.73	33.94	39.12	77.48

Source: Authors, based on MICS data

Table 5 shows results on HOI on selected nutrition indicators by countries. HOI for the selected nutrition indicators in Congo DR, Guinea Bissau and Mali were 47.94%, 61.40% and 53.63%, respectively.

**Table 5:** Human Opportunity Index on Selected Nutrition Indicators by countries

		NUTRI1 (%)	Stunting (%)	Wasting (%)	Underweight (%)	Blood tests (%)
**Coverage (C)**	**Democratic Republic of Congo**	51.87	19.01	5.59	16	27.34
	**Guinea Bissau**	65.15	19.26	5.14	11.17	36.72
	**MALI**	58.29	14.87	11.62	16.31	25.16
**Penalty (P)**	**Congo DR**	3.93	1.07	0.46	1.62	4.89
	**Guinea Bissau**	3.75	2.24	1.23	2.31	4.72
	**MALI**	4.66	1.50	0.93	1.53	3.27
**Dissimilarity (D)**	**Congo DR**	7.57	5.64	8.27	10.15	17.90
	**Guinea Bissau**	5.76	11.64	23.91	20.71	12.86
	**MALI**	7.99	10.07	8.03	9.38	12.98
**Equality (E)**	**Congo DR**	92.43	94.36	91.73	89.85	82.1
	**Guinea Bissau**	94.24	88.36	76.09	79.28	87.14
	**MALI**	92.01	89.93	91.97	90.62	87.02
**HOI**	**Congo DR**	47.94	17.94	5.13	14.37	22.45
	**Guinea Bissau**	61.40	17.02	3.91	8.85	32
	**MALI**	53.63	13.37	10.68	14.78	21.90

Source: Authors, based on MICS data

### Circumstances and access to healthcare and nutrition services

#### Access to healthcare services

**Fig. 1:**
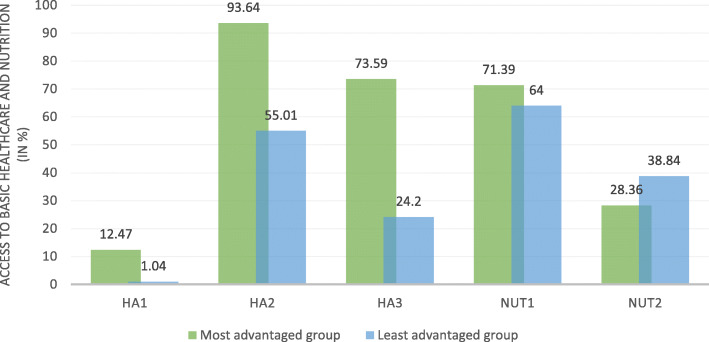
Access to basic healthcare and nutrition by groups in Guinea Bissau

The results obtained vary according to the country and the healthcare indicators used for the children of each group. In Guinea Bissau, the results (Fig. 1) show a significant disparity in access to health services between the children of the two groups. It appears that children belonging to the most favorable group are those who benefit much more from access to health services with higher access rates for immunization (93.64%) and water and sanitation facilities (73.59%). The rate of access to healthcare services before and after delivery, although higher among children in the most favorable group, remains at a very low level for both groups (14.47 and 1.04% respectively). It is about 12.47% for children in the most favorable group against 1.04% for those in the least favorable group.

**Fig. 2:**
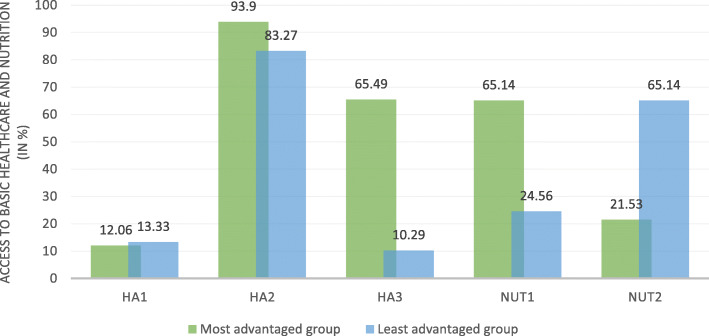
Access to basic healthcare and nutrition by groups in Congo DR

In Congo DR, Fig. 2 shows disparity in access to healthcare services between the children of the two groups with high gap in access to water and sanitation facilities (55.20%). The access rate is high for immunization. It is about 93.9% for children in the most favorable group against 83.27% for children in the least favorable group. For access to healthcare services before and after delivery, the rate is very low for the two groups.

**Fig. 3:**
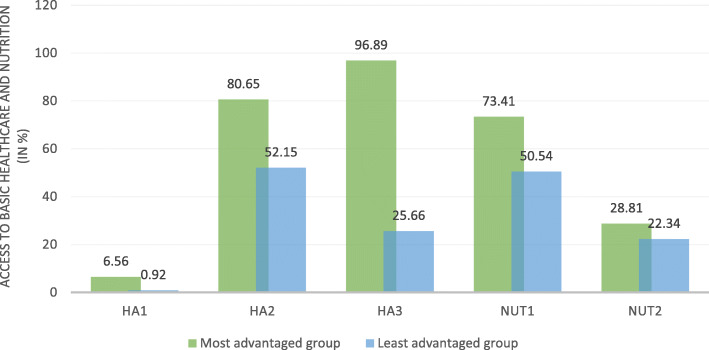
Access to basic healthcare and nutrition by groups in Mali

Figure 3 shows wide disparities in access to healthcare services between children of the two extreme groups in Mali. While access rates are higher for children in the most favorable group, it remains very low for both groups when considering access to healthcare services before and after delivery. These access rates stand at 6.56% for children in the most favorable group and 0.92% for those in the least favorable group.

#### Access to nutrition services

Contrary to the observation made on access to healthcare services in Guinea Bissau, the disparities are not very pronounced between the children of the two groups in terms of access to nutrition outcome (Fig. 1). The propensity of not having a malnutrition problem is high for children of the most favorable group while the access of the mother to blood tests during the pregnancy period is high for children of the least favorable group. The differences are established at 7.39% for the propensity of not having a malnutrition problem and 10.48% for the access of the mother to blood tests during the pregnancy period between the children of the two extreme groups. Figure 2 shows very significant disparities between the children of the two groups in terms of access to nutrition outcome in Congo DR contrary to the situation observed in Guinea Bissau. Similarly, to the case of Guinea Bissau, the propensity of not having a malnutrition problem is high for children of the most favorable group (with a gap of 40.58%), while the access of the mother to blood tests during the pregnancy period is high for children of the least favorable group (with a gap of 43.61%).

There are also nutritional disparities in Mali (Fig. 3). The two nutritional status indicators have a higher access rate for children in the favorable group in Mali, contrary to the situation in Guinea Bissau and Congo DR. The gap is larger for the propensity of not having a malnutrition problem (about 22.87%) and smaller for the access of the mother to blood tests during the pregnancy (6.47%).

### Inequality of opportunity in access to healthcare facilities and basic nutrition

#### Inequality of opportunity in access to basic healthcare

**Fig. 4:**
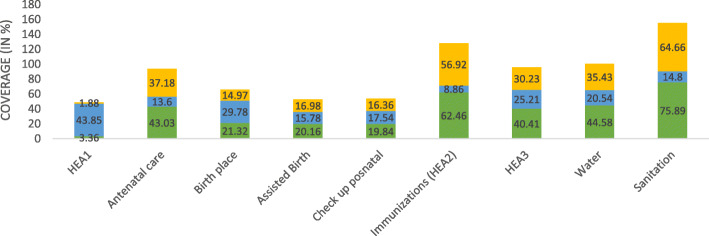
Inequality of opportunity in access to healthcare services in Guinea Bissau

The results in Fig. 4 reveal that in Guinea Bissau, inequality of opportunity is important in access to health services before and after delivery (43.85%). This is accompanied by very low levels of HOI and the coverage rate (1.88% and 3.36% respectively).

**Fig. 5:**
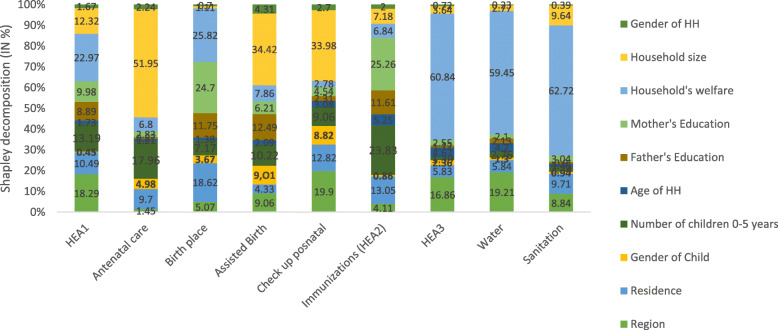
Shapley decomposition of healthcare opportunities in Guinea Bissau

However, the situation is a little different when considering immunization and access to basic housing services for which there are relatively high levels of HOI (56.92% and 30.23%) and the coverage rate (62.46% and 40.41%). However, it should be noted that the HOI attained 64.66%, indicating that a high number of children benefit from sanitation services regardless of their living conditions. Shapley’s decomposition reveals that it is the household’s welfare that contributes much more to inequality in access to basic housing services and access to health services before and after delivery (60.84% and 22.97% respectively). For access to immunization, it is the mother’s education level which contributes more to the inequality (25.26%) (Fig. 5).

**Fig. 6:**
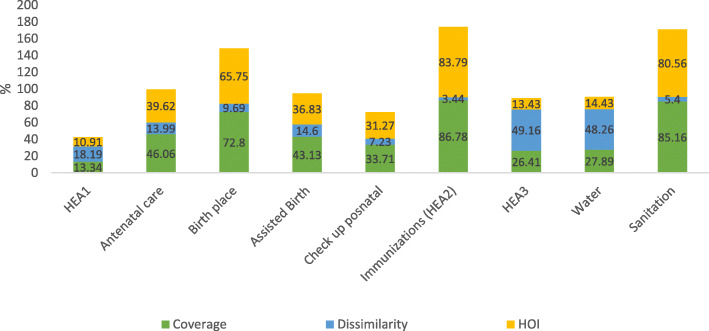
Inequality of opportunity in access to healthcare services in Congo DR

In Congo DR, the IoP measured by HOI index is only high for the immunization composite indicator (83.79%) (Fig. 6).

**Fig. 7:**
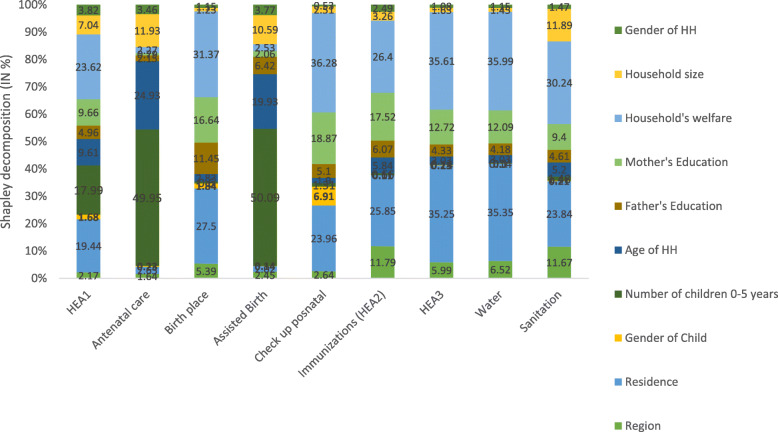
Shapley decomposition of healthcare opportunities in Congo DR

For the other composite indicators (HEA1 and HEA3), the IoP appears to be greater than the HOI and the coverage rate. The IoP is more pronounced for access to basic housing services (49.16%). The Shapley decomposition shows that household welfare is the circumstance variable which contributes much more to the inequalities observed for the three composite indicators of access to health (HEA1–23.62%, HEA2–26.4% and HEA3–35.61%) in Congo DR (Fig. 7).

**Fig. 8:**
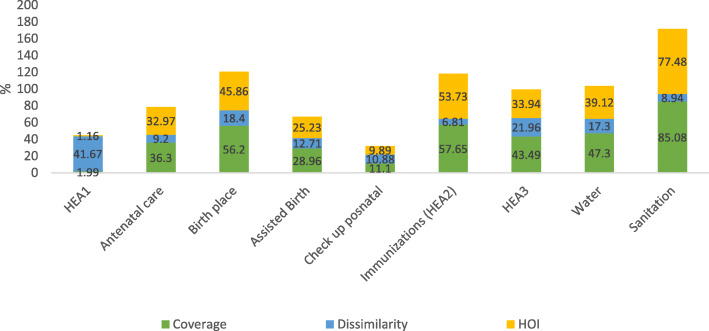
Inequality of opportunity in access to healthcare services in Mali

Figure 8 shows that in Mali, IoP (D-index) is higher for access to health services before and after delivery (41.67%) than for other composite indicators (HA2 and HA3). For the latter, the HOI (53.73% and 33.94%) and the coverage rate (57.65% and 43.49%) are higher than the level of the dissimilarity index.

**Fig. 9:**
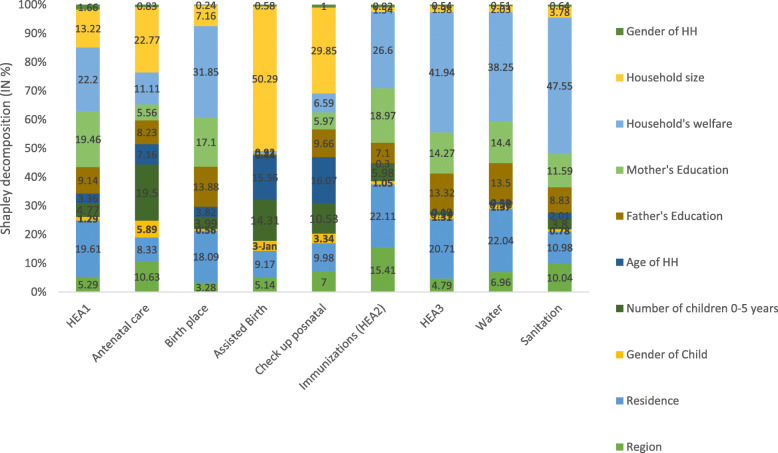
Shapley decomposition of healthcare opportunities in Mali

Similarly, in Mali, the Shapley decomposition (Fig. 9) reveal that the household welfare contributes much more to the inequalities in the three composite indicators of access to healthcare (HEA1–22.2%, HEA2–26.6% and HEA3–41.94%).

#### Inequality of nutritional opportunity

**Fig. 10:**
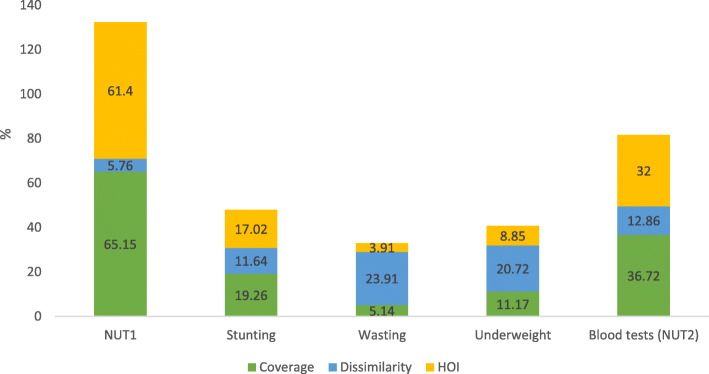
Inequality of opportunity in access to nutrition and its decomposition in Guinea Bissau

IoP in Guinea Bissau is low for the propensity of not having a malnutrition problem (about 5.76% for NUT1) and for access of the mother to blood tests during the pregnancy (12.86% for NUT2) in comparison to the respective HOI levels and the coverage rate (Fig. 10). IoP becomes more important when considering indicators such as wasting (23.91%) and underweight (20.72%).

**Fig. 11:**
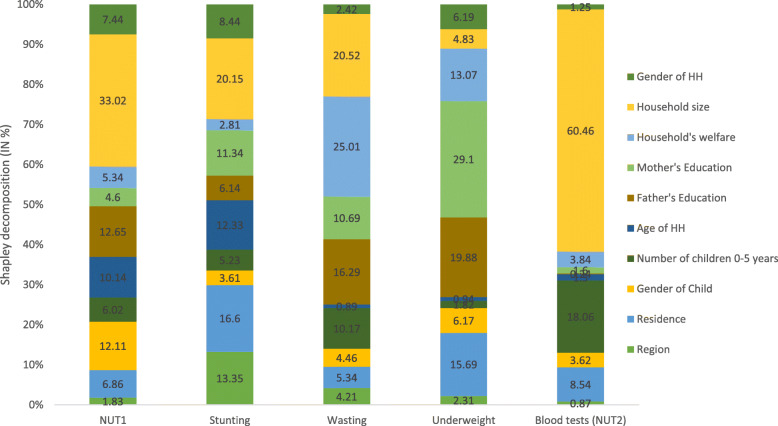
Shapley decomposition of nutrition opportunities in Guinea Bissau

According to the Shapley decomposition (Fig. 11), the household size is the circumstance variable which explains much more the inequality observed in the propensity of not having a malnutrition problem (33.02%) and the access of the mother to blood tests during the pregnancy (60.46%).

**Fig. 12:**
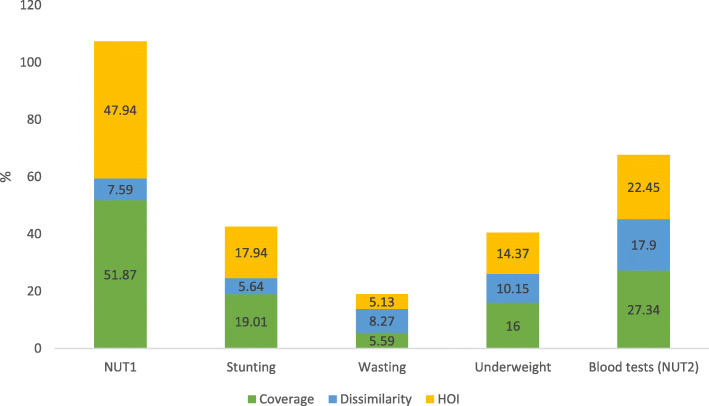
Inequality of opportunity in access to nutrition and its decomposition in Congo DR

Figure 12 shows results almost similar to those obtained in Guinea Bissau but with slightly higher levels in Congo DR. IoP is about 7.59% for the propensity of not having a malnutrition problem against 17.9% for the access of the mother to blood tests during the pregnancy.

**Fig. 13:**
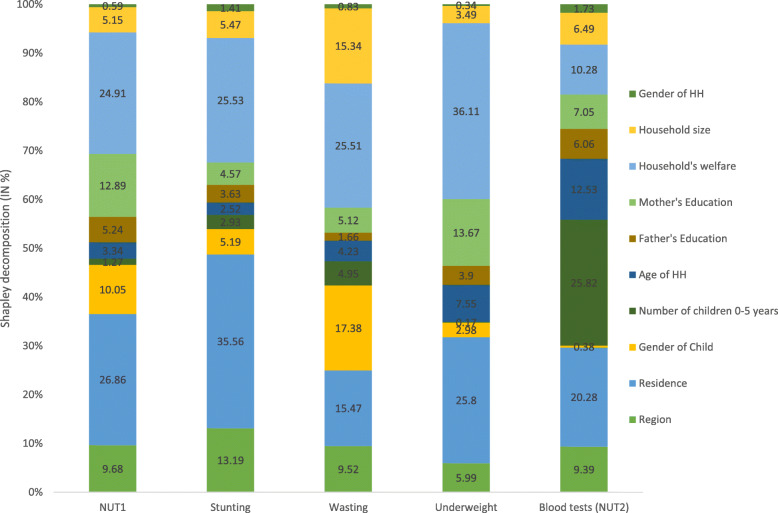
Shapley Decomposition of Nutrition Opportunities in Congo DR

Figure 13 shows that place of residence contributes much more to inequality in the propensity of not having a malnutrition problem (26.86%) and the number of children under five explains more of access of the mother to blood tests during pregnancy (25.82%).

**Fig. 14:**
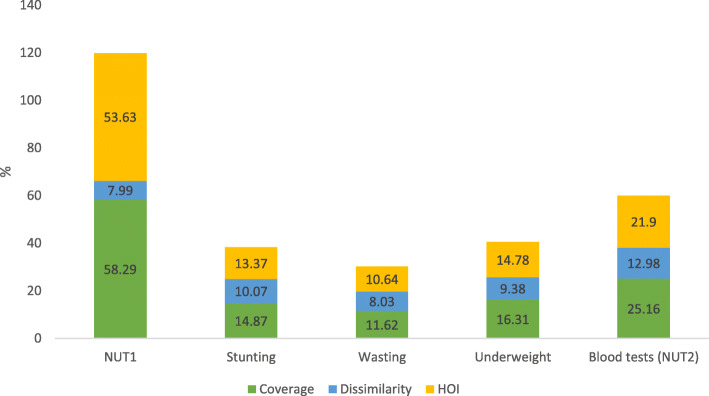
Inequality of Opportunity in Access to Nutrition and its decomposition in Mali

The results obtained in Mali (Fig. 14) are also close to those obtained in the other two countries in terms of the low level of inequality in the propensity of not having a malnutrition problem (about 7.99% for NUT1) and for access of the mother to blood tests during the pregnancy (12.98% for NUT2). HOI levels and the coverage rate are high for the propensity of not having a malnutrition problem (about 53.63% and 58.29% for NUT1).

**Fig. 15:**
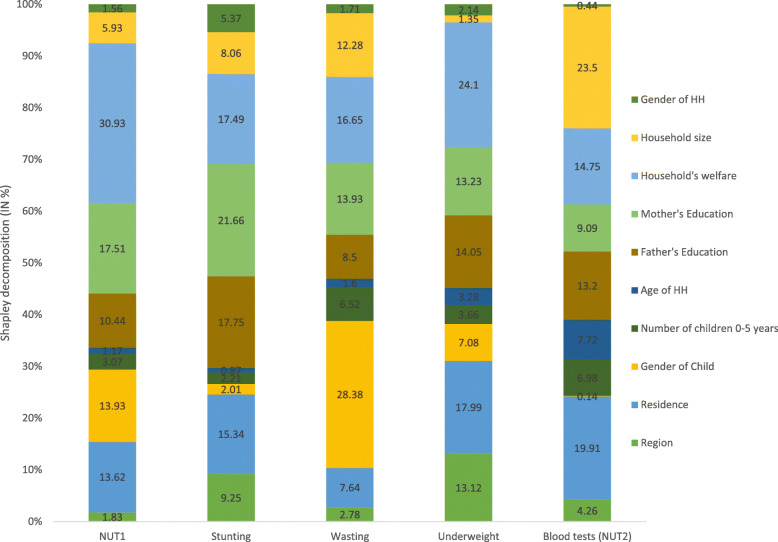
Shapley Decomposition of Nutrition Opportunities in Mali

Figure 15 reveals that household’s welfare and household size contribute respectively much more to inequality in the propensity of not having a malnutrition problem (30.93%) and inequality in the mother to blood tests during the pregnancy (23.5%).

## Discussion

Our study focused on the patterns and extent of IoP in health and nutrition among children under-five across three countries in sub-Saharan Africa with low HDI and the estimate of the relative contribution of circumstances that are beyond the control of children under-five using data from MICS. In general, our findings revealed that there are disparities in access to children’s health and nutrition outcome and that, the IoP is a reality in the three countries studied. The main results obtained vary according to the countries and the indicators used for health and nutrition outcome. However, the inequalities of opportunity observed between the children of the most favorable group and those of the least favorable group, remain in general at significant levels and calls on government of these countries to implement policies taking them into account.

With the two extreme sub-groups (the most and the least favorable group) computed for the three countries, the results showed a significant disparity in access to health services between children of the two groups. It appears that children belonging to the most favorable group are those who have higher access to health services (with the exception of access to health services before and after delivery in Congo DR), immunization (from 80.65 to 93.9% depending on the country) and water and sanitation facilities (from 65.49 to 96.89% depending on the country). Considering that children’s access to most health services is indirect and often passes through mothers, our results follow the same logic as those which find that parent (mothers in particular) living in good (favorable) conditions were associated with better access to healthcare services [33, 34]. Other studies show that poor living conditions are associated with poor access to basic healthcare services [13, 35].

The situation is similar in relation to access to children’s nutrition for which there are disparities linked to differences in terms of living conditions between the two groups for the three countries. The extent of these disparities is less pronounced in Guinea Bissau compared to the other two countries [36]. These disparities in children’s nutrition outcome according to socio-demographic is also found in the literature on inequality of health opportunity [23]. These results suggest that efforts in promoting, independently of the socio-economic and demographic characteristics of households, the major part of the services necessary for the health of the mother during pregnancy in order to fight against child malnutrition are much more noticeable in Guinea Bissau than the other two countries. It should be noted that, except Mali, the propensity of not having a malnutrition problem is high for children of the most favorable group, while the mother’s access to blood tests during the pregnancy period is high for children of the least favorable group. This results can be explained by the fact that mothers with the characteristics of the least favorable group are much more sensitive to this aspect of health care in order to fight against malnutrition.

The investigation of the inequality of opportunity in order to understand these disparities observed in the access to health and nutrition outcome between the children of the two extreme groups in relation to the variables of the circumstances reveals that IoP vary according to the three countries and healthcare and nutrition outcomes. It appears that, in Guinea Bissau and Mali, IoP is important in access to healthcare services before and after delivery (43.85% and 41.67% respectively) and in access to basic housing services for Congo DR (49.16%). For all the three countries, the results indicate that IoP has a significant contribution to disparities observed in child healthcare outcome. This finding is consistent with previous studies on the measure of children’s health IoP [22, 37]. These high levels of IoP are associated with relatively low levels of HOI and the coverage rate reflecting the fact that only a small fraction of these indicators is equitably distributed between the children, independently of circumstances in which they live in these countries. However, the situation is different when considering immunization for which there are relatively high levels of HOI ranging from 53.73 to 83.79% and the coverage rate ranging from 57.65 to 86.78% depending on the country. This indicates that a high number of children benefit from immunization services regardless of their living conditions in these countries.

Shapley’s decomposition shows that the household welfare is the circumstance variable which contributes much more to the inequalities observed for the three composite indicators of access to health (HA1, HA2 and HA3) in Congo DR and Mali. The situation is similar for access to basic housing services and access to healthcare services before and after delivery in Guinea Bissau but for access to immunization, it is the mother’s education level which contributes more to the inequality. Our finding is in line with previous studies which have also shown that household wealth and mother’s education level are among the socio-economic characteristics that contribute the most to inequality [23, 37, 38].

The levels observed for the D-index show that the inequalities of opportunity are a reality in these countries and must be considered considerably in interventions to reduce inequality because it affects seriously the access to basic housing services which are important aspects of government interventions, aiming to ensure child health and survival, development and growth.

Compared to the case of access to healthcare services, inequality of nutritional opportunity in these countries is at low levels ranging from 5.76 to 7.99% for the propensity of not having a malnutrition problem and from 12.86 to 17.90% for mother’s blood tests during pregnancy depending on the country. However, the respective HOI levels and the coverage rate are relatively high meaning that efforts are made to reduce inequality of nutritional opportunity. Despite the low levels generally observed for propensity of not having a malnutrition problem and for the access of the mother to blood tests during the pregnancy, the results show that the prevalence of stunting, wasting and underweight depend on life circumstance variables. Our result is in line with those studies which find that the low values of IoP can be considered as a lower bound estimate of the circumstance’s variables selected and must be considered in policies to reduce inequalities. One of these studies focused on Arab world and Turkey [22] and the authors found that IoP varies between 4% and 18% depending on the country.

According to the Shapely decomposition, while household’s welfare is the circumstance variable which explains much more the inequality observed in the propensity of not having a malnutrition problem in Guinea Bissau and Mali, it is the place of residence which contributes much more in Congo DR. These results are also consistent with those which, in addition to the household wealth and mother’s education, mention the place of residence as an important characteristic in explaining the IoP [37–39]. For mother’s blood tests during the pregnancy, household’s welfare, number of children under five [23] and household size are circumstance’s variables which explained much more inequality respectively in Guinea Bissau, Congo DR and Mali.

## Conclusion

The study findings identify the disadvantaged populations and the determinants that limit children’s development in early childhood. The disparities observed can be explained by the household’s welfare, the mother’s education level, the place of residence, the number of children under-5 and the size of the households. The household’s welfare is the circumstance variable which contributes much more to inequality in access to healthcare and nutrition services among children of the most favorable group and those of the least favorable group. With gaps in child health and nutrition arising in childhood, identification of inequalities will inform policy interventions aiming to fill these gaps and to improve equal opportunity indices for children in sub-Saharan Africa. Ultimately, the findings will help to steer the life course of disadvantaged children towards healthy and productive lives in adulthood. These results also imply that interventions aimed at improving well-being or reducing poverty can help to reduce considerably IoP. In addition, promoting the education of women with considerations on the place of residence in which they live can be an effective intervention to reduce the IoP.

## Abbreviations

DHS: Demographic and Health Survey
HA1: Access to healthcare services before and after birth.
HA2: Access to immunizations
HA3: Access to housing services (water and sanitation facilities)
HOI: Human Opportunity Index
IOP: Inequality of Opportunity
NUT1: Propensity of not having a malnutrition problem during the 5 years after birth
NUT2: Access of the mother to blood tests during the pregnancy period
